# Fabrication of Three-Dimensionally Deformable Metal Structures Using Precision Electroforming

**DOI:** 10.3390/mi13071046

**Published:** 2022-06-30

**Authors:** Seitaro Kumamoto, Souichiro Fukuyama, Seiya Nagano, Keiichiro Yasuda, Yusuke Kitamura, Masaaki Iwatsuki, Hideo Baba, Toshihiro Ihara, Yoshitaka Nakanishi, Yuta Nakashima

**Affiliations:** 1 Graduate School of Science and Technology, Kumamoto University, 2-39-1 Kurokami, Chuo-ku, Kumamoto 860-8555, Japan; s.kumamoto@ogic.ne.jp (S.K.); souichirof1@yahoo.co.jp (S.F.); seiya-nagano@tamadic.co.jp (S.N.); 2 Ogic Technologies Co., Ltd., 2-9-9 Kamikumamoto, Nishi-ku, Kumamoto 860-0079, Japan; kyasuda@ogic.ne.jp; 3 Faculty of Advanced Science and Technology, Kumamoto University, 2-39-1 Kurokami, Chuo-ku, Kumamoto 860-8555, Japan; ykita@kumamoto-u.ac.jp (Y.K.); toshi@chem.kumamoto-u.ac.jp (T.I.); y-naka@mech.kumamoto-u.ac.jp (Y.N.); 4 Faculty of Life Science, Kumamoto University, 1-1-1 Honjo, Chuo-ku, Kumamoto 860-8556, Japan; maiwa217@kumamoto-u.ac.jp (M.I.); hdobaba@kumamoto-u.ac.jp (H.B.); 5 Institute of Industrial Nanomaterials, Kumamoto University, 2-39-1 Kurokami, Chuo-ku, Kumamoto 860-8555, Japan; 6 International Research Organization for Advanced Science & Technology, Kumamoto University, Kumamoto 860-8555, Japan; 7 Fusion Oriented Research for Disruptive Science and Technology Researcher (FOREST Researcher), Japan Science and Technology Agency, Saitama 332-0012, Japan

**Keywords:** precision processing, electroforming, MEMS, metal structures, deformation

## Abstract

It is difficult to fabricate three-dimensional structures using semiconductor-process technology, because it is based on two-dimensional layered structure fabrication and the etching of thin films. In this study, we fabricated metal structures that can be dynamically deformed from two-dimensional to three-dimensional shapes by combining patterning using photolithography with electroforming technology. First, a resist structure was formed on a Cu substrate. Then, using a Ni sulfamate electroforming bath, a Ni structure was formed by electroforming the fabricated resist structure. Finally, the resist structure was removed to release the Ni structure fabricated on the substrate, and electroforming was used to Au-plate the entire surface. Scanning-electron microscopy revealed that the structure presented a high aspect ratio (thickness/resist width = 3.5), and metal structures could be fabricated without defects across the entire surface, including a high aspect ratio. The metallic structures had an average film thickness of 12.9 µm with σ = 0.49 µm, hardness of 600 HV, and slit width of 7.9 µm with σ = 0.25 µm. This microfabrication enables the fabrication of metal structures that deform dynamically in response to hydrodynamic forces in liquid and can be applied to fields such as environmental science, agriculture, and medicine.

## 1. Introduction

In fields such as electronic-equipment manufacturing, biotechnology, and medicine, miniaturization and large-scale integration of systems are advancing, and process technology is being used to upgrade the functions of materials, substrates, and microdevices [1,2,3,4,5,6]. This development includes the demand for various structures with complex shapes, such as three-dimensional structures for realizing effective microregional chemical reactions, mixing, and analyses in various fields, including biological microelectromechanical systems (bioMEMSs) and micro total analysis systems (µTASs) [7,8,9,10,11]. Studies on the fabrication of simple 2.5-dimensional structures with metals have been conducted, by devising methods for laminating and tapering to achieve these structures. However, these metal structures are static. Dynamic devices include structures in which polymers such as polydimethylsiloxane deform upon the application of pressure [12] and movable structures such as propellers that employ photopolymerization reactions [13]. However, although resin materials deform dynamically, it is impossible to perform various chemical modifications on structural surfaces. Single-crystal silicon can be combined with multiple materials to form a three-dimensional structure, but it is brittle. Although three-dimensionally deformable materials include comb actuators used in silicon material, chemical modification of the surfaces cannot be performed. In contrast, precision processing, such as cutting and grinding, may be used to produce free three-dimensional shapes for metal materials. However, such processing can cause variations by as much as 10 µm [14,15,16], which is unsuitable when the required precision is on the order of microns. One option for processing that can be realized with high precision and a high aspect ratio is deep reactive ion etching (DRIE) of silicon [17,18,19,20], used in anisotropic processing. DRIE is widely employed for producing dynamic random-access memories (DRAMs) and other devices. However, the cost of fabrication is a problem, as expensive equipment and time are required to achieve the necessary degree of vacuum. In comparison, high aspect ratios can be fabricated easily, as the combined photolithographic patterning and electroforming technique does not require a high vacuum or expensive equipment. However, the use of electroforming technology requires a seed-layer-forming process, which poses problems for productivity. The Lithographie, Galvanoformung, and Abformung (LIGA) process [21,22] is utilized when microelectromechanical system (MEMS)-level microfabrication techniques are required. However, X-ray equipment is expensive, posing problems for productivity and simplicity [23]. Precision electroforming technology, which combines photolithography and electroforming technology, has the advantages of better dimensional accuracy and higher productivity, compared with machining approaches such as cutting and grinding, and enables high-speed replication of the parent mold [24]. However, most extant studies on robust structures for miniaturization and precision improvement are concerned with fabricated structures [25,26,27,28], whereas those on structures with three-dimensional deformability are lacking. The microstructure accuracy has been reported to be limited to a 10% variation in a 50 µm design [29]. If a method with a higher aspect ratio and accuracy could be developed, microfabrication technology could be improved, and precision components could be produced. There are some examples of using precision electroforming technology to fabricate electric-shaver blades, but the structures are robust, require a hand-pushing force, and do not exhibit chemical modification. If a structure that deforms from a two-dimensional shape into a three-dimensional shape due to the hydrodynamic force could be produced via precision electroforming, the scope of the microdevice application (adaptation) would be expanded in a useful manner. μTAS has the advantage of reducing the amount of liquid required and shortening the analysis time by using a small amount of liquid. In microfluidic systems, the flow rate is approximately 1 µL/min^−1^ mL/min [30,31,32]. The device must be agitated because it is difficult to mix the liquid when the width and depth of the flow channel decrease, the Reynolds number decreases, and the flow becomes laminar. For example, it would be advantageous to create a structure that dynamically agitates the inside of a microchannel to enable effective mixing. Furthermore, if it can dynamically deform into a three-dimensional structure or agitate, it can be applied to cases in which the liquid is fed in fixed quantities or filtered according to the flow rate. Therefore, in this study, we focused on the use of precision electroforming technology to investigate a method of fabricating an original metal structure capable of three-dimensional elastic deformation in response to a hydrodynamic force. Although flat-shaped structures can be fabricated using semiconductor processes, this method can produce structures that deform three-dimensionally by a hydrodynamic force. Furthermore, we evaluated its physical properties and basic operations.

## 2. Experimental Method

### 2.1. Design of a Three-Dimensional Elastically Deformable Metal Structure

**Figure 1:**
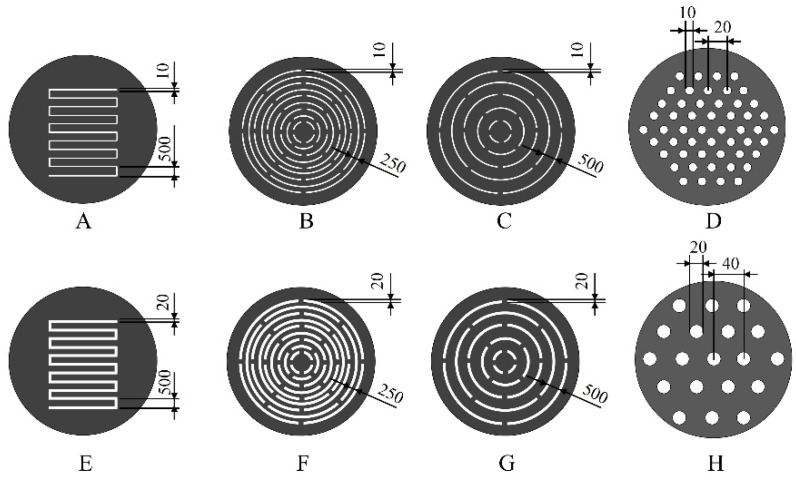
Overview of metal structure designs. The metal structures were designed with diameters of 15 mm and slit widths of 10 µm and 20 µm. Types A and E are comb-shaped, and Types B, C, F, and G are structures with multiple slits arranged in an arc shape. Types A–C and E–G have elastic structures and are designed to deform into three-dimensional shapes due to hydrodynamic force. The reason for utilizing different slit widths of 10 μm and 20 μm was to confirm that the thin film could be fabricated as the designed structure. The structures with fine pores on flat plates in Types D and H were fabricated to demonstrate the designs that needed to be devised.

Figure 1 presents the designs of the eight types of fabricated metallic structures. Types A and E consist of multiple slits arranged in comb-like shapes. The total length of the slits is approximately 6 mm, and the slit widths in Types A and E are 10 µm and 20 µm, respectively. In these structures, the root portion of the comb teeth is deformed at its origin by hydrodynamic forces, and plate-like structures mutually intersect. Types B and C are structures in which multiple slits are arranged in an arc shape, the slit width is 10 µm, and the pitches are 250 µm and 500 µm, respectively. Types F and G are similar structures wherein multiple slits are arranged in an arc shape, the slit width is 20 μm, and the pitches are 250 µm and 500 µm, respectively. In structures with these arc-shaped slits, the slit portions are deflected by the fluid force received and deform into shapes similar to that of an insect net. Metallic structures that deform into three-dimensional shapes of Types A–C and E–G have elastic structures and are designed to return to their original shapes when the hydrodynamic force is unloaded, even when deformed in response to hydrodynamic forces. Types D and H are structures with fine pores on flat plates, having diameters of 10 µm and 20 µm and pitches of 20 µm and 40 µm, respectively. These structures were fabricated to demonstrate that the three-dimensional deformation of a metal structure requires machining and design ingenuity.

### 2.2. Methods for Evaluating Physical Properties of Metal Structures

The stiffness of the fabricated material must be adjusted to fabricate a three-dimensionally deformable metal structure. It is important to consider the stiffness of the Ni structure for device design and performance, as it changes the shape maintenance and deformation capacities of the Ni structure. In addition, the film thickness, hardness, and slit width must be homogeneous. In Ni electroforming, the current is concentrated at the edge and outer peripheral portion of the substrate, owing to the current distribution problem, resulting in a thicker film [33]. Therefore, the film-thickness variation within the substrate plane was evaluated with four different distances, d = 24, 48, 60, and 84 mm, between the cathode and anode. The film thickness was measured at 89 locations within the substrate surface using a fluorescent X-ray film-thickness meter (SFT-9400, SII Nanotechnology Co., Ltd., Chiba, Japan). Furthermore, to evaluate the stiffness of the metal structures, the hardness of four types of metal structures was measured by adjusting the material with a film thickness of 40 μm. For the hardness measurements, a microhardness tester (HM-221, Mitutoyo Corporation, Kawasaki, Japan) was used to obtain measurements at nine points under a load of 0.49 N and a loading time of 10 s for determining the mean value. Furthermore, for the dimensions of the slit width of the fabricated metallic structure, a digital microscope (VHX-6000, KEYENCE Co., Ltd., Osaka, Japan) was used to measure the 12 in-plane locations for evaluating the variations.

### 2.3. Metal Structure Fabrication Method

**Figure 2:**
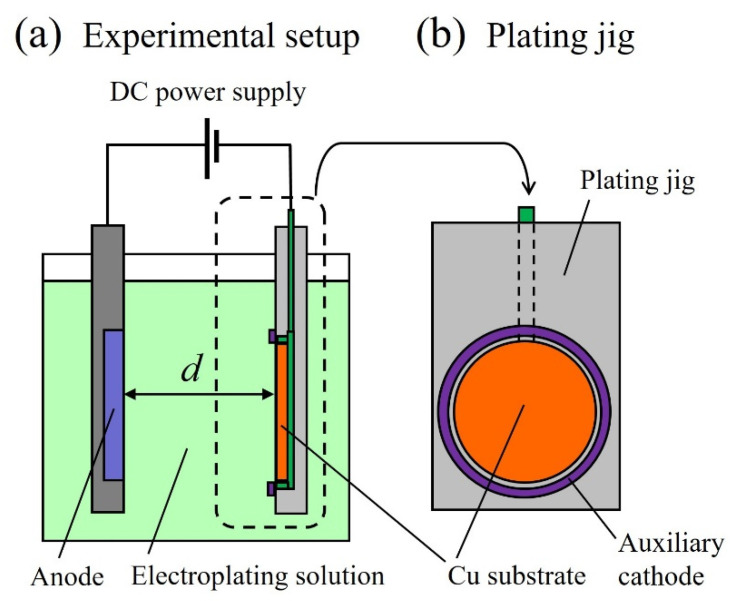
(**a**) is Experimental setup for electroforming. A Cu plate was used for the cathode, and a Ni anode plate containing sulfur was used for the anode, while d is the distance between the cathode and anode. (**b**) is Plating jig and the auxiliary cathode was installed at the periphery of the facility to ensure uniform film thickness.

Figure 2 shows the electroforming apparatus used to fabricate the metal structures. A Cu plate for a 4-inch silicon wafer (Yamamoto Plating Tester Co., Ltd., Tokyo, Japan) was employed as the cathode, and a Ni anode plate containing sulfur (Yamamoto Plating Tester Co., Ltd.) was utilized as the anode. A YPP-15,031 (Yamamoto Plating Tester Co., Ltd.) was used as the rectifier for the electroforming. Table 1 lists the basic configuration of the electroforming bath used in this study; the concentration of nickel sulfamate tetrahydrate in the bath (nickel sulfamate 900, JX Metal Shoji Co., Ltd., Tokyo, Japan) was 600 g/dm^3^. The boric acid in the bath (Nacalai Tesque Inc., Kyoto, Japan) was prepared at a concentration of 30 g/dm^3^. The pH of the electroforming bath was 4.0 ± 0.1, and the temperature of the electroforming bath was 40 ± 2 °C. The temperature was controlled by a temperature-control unit. The anode and cathode areas were 165 cm^2^ and 136 cm^2^, respectively. Ni dissolves on the anode side and Ni is deposited on the substrate on the cathode side. Moreover, Ni metal is deposited, and a Ni electroformed structure is formed. The chemical equations for the anode and cathode sides are shown in Equations (1)–(4) and (5)–(6), respectively.

Ni → Ni^2+^ + 2e^−^ (1)

Ni + H_2_O → NiO + 2H^+^ + 2e^−^ (2)

3Ni + 4H_2_O → Ni_3_O_4_ + 8H^+^ + 8e^−^ (3)

2H_2_O → O_2_ + 4H^+^ + 4e^−^ (4)

Ni^2+^ + 2e^−^ → Ni (5)

2H^+^ + 2e^−^ → H_2_ (6)

## 3. Results and Discussion

### 3.1. Evaluation of Fabricated Substrate in-Plane Film Thickness

**Figure 3:**
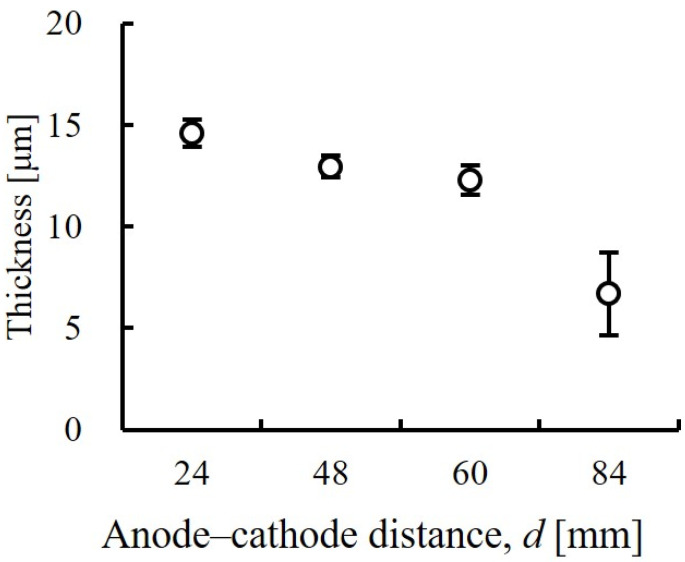
Evaluation of variations in Ni-coating thickness. The inter-electrode distance during Ni electroforming was set to four values, and the film thickness was measured by performing processing for 10 min at a target film thickness of 10 μm. The average film thickness of 12.9 μm and σ = 0.49 μm minimized the variation at an interelectrode distance of 48 mm. As the coating thickness is proportional to the electroforming time, if the variation is small, the desired film thickness can be obtained via time adjustment.

**Figure 4:**
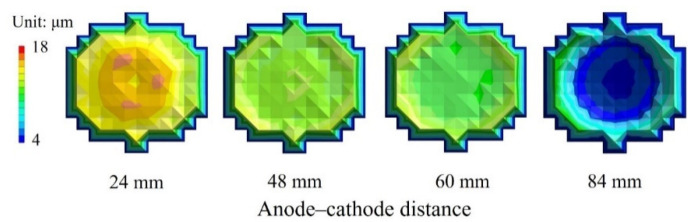
Substrate in-plane-coating thickness distribution. The average film thickness is 12.3 μm and σ = 0.72 μm, with a 60 mm inter-electrode distance, and at 48 mm, the variation is minimized, with an average film thickness of 12.9 μm and σ = 0.49 μm. There is almost no difference in the substrate in-plane film thickness with a 48 mm inter-electrode distance, and the variation is the smallest in this case.

To evaluate the in-plane film thickness variation of the substrate produced by Ni electroforming, we measured the film thickness by setting a target film thickness of 10 µm and processing it for 10 min. Subsequently, the inter-electrode distance was set to the four aforementioned distances, d = 24, 48, 60, and 84 mm. Figure 3 shows the relationship between the inter-electrode distance and the electron-coating thickness. The longer the inter-electrode distance, the thinner is the film produced by Ni electroforming, and the average film thickness is the smallest at 6.7 µm with d = 84 mm. The variations are at their smallest with d = 48 mm, with an average film thickness of 12.9 µm and a standard deviation σ of 0.49 µm. Figure 4 shows the film-thickness distribution at each interelectrode distance. Negligible differences are observed in substrate in-plane film thickness, and the film-thickness variation is the smallest when d = 48 mm. With electroforming, the current is concentrated in the outer peripheral portion of the substrate when the distance between electrodes is large, and the current concentration in the central portion is high when the inter-electrode distance is small [34]. This suggests that d = 48 mm is suitable for achieving uniform film thickness. For d = 24, 48, and 60 mm, film thickness was more than 10 μm because only a small amount of electroforming was deposited on the auxiliary cathode installed at the periphery of the facility to ensure uniform film thickness, and the inside of the substrate became thick. Furthermore, because the electroforming is proportional to the electroforming time [35,36], if the throwing power is good, the desired film thickness may be expected. Based on these results, to suppress film-thickness variation in the various metal structures arranged on the substrate, we applied d = 48 mm in the subsequent evaluation. This is because the resulting coating thickness at the outermost peripheral portion being thin shows the interpolated numerical values of the unplated area and adjacent portion at the electroforming contact point, so can be ignored.

### 3.2. Evaluation of Stiffness of Metal Structures

**Figure 5:**
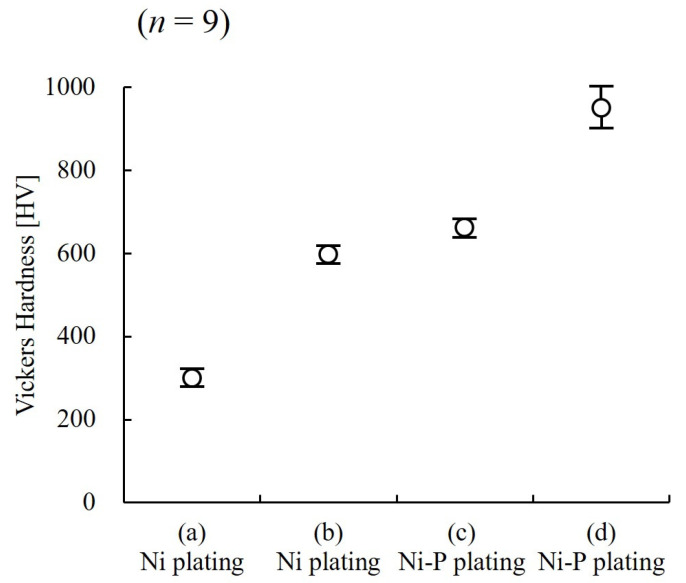
Ni-coating-hardness measurement results. Sample (**a**) is a Ni coating obtained from a basic bath, (**b**) is a coating adjusted by adding 5 mL/L [40] of sulfurous additive to the basic bath, (**c**) is a coating adjusted by adding 20 g/L of phosphorus acid to the basic bath, and (**d**) is a coating of the sample (**c**) heat-treated at 350 °C for 1 h. Sample (**b**) has a hardness of 600 HV and achieves stiffness allowing elastic deformation.

**Figure 6:**
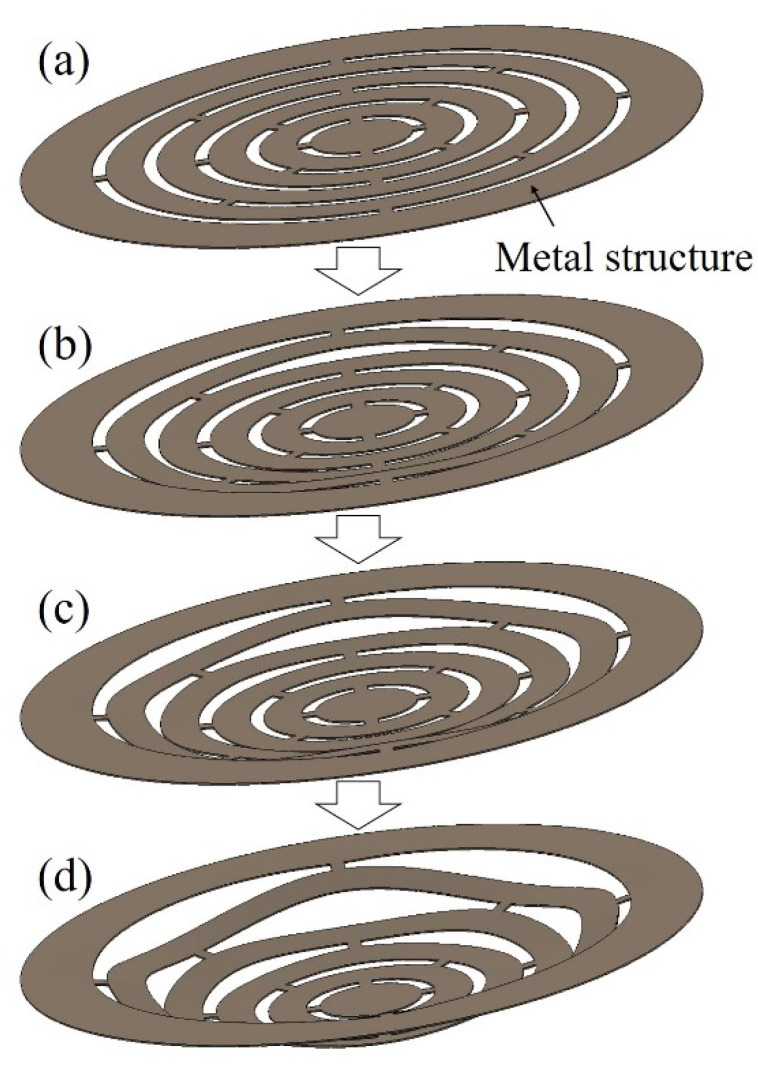
Schematic of deformation of metal structures due to hydrodynamic forces. (**a**) Initial state of the metal structure. (**b**) When the hydrodynamic forces are small, the deformation of the metal structure is small. (**c**,**d**) The greater the hydrodynamic forces is, the greater the deformation of the metal structure.

Figure 5 shows the hardness measurement results for the four types of samples, to evaluate the stiffness of the Ni structure. Sample (a) is a Ni coating obtained from the basic bath in Table 1, (b) is a coating adjusted with a high-hardness additive, (c) is a Ni–P alloy coating, and (d) is a sample of Ni–P coating heat-treated at 350 °C for 1 h. Compared to the Ni films in samples (a) and (b), the Ni–P alloy films in samples (c) and (d) are harder, especially in the sample (d), which was heat-treated and had a high Vickers hardness of 950 HV. This high hardness is thought to be due to crystallization [37,38] of the Ni–P alloy by heat treatment. However, we found that the samples (c) and (d) were much more difficult to deform than the other samples [39], and the coating cracked from simple bending, making them unsuitable as three-dimensional elastically deformable structures (Supplementary Materials Figure S1). Further, while sample (a) was easily deformed at approximately 300 HV, good stiffness with 600 HV hardness and elastic deformation were achieved when the plated coating was adjusted with a high curing additive in the sample (b); hence, we used this sample in subsequent evaluations. The Young’s modulus of the sample (b) was 200 GPa. Figure 6 shows a schematic of the deformation expected when a metallic structure is subjected to hydrodynamic forces. When a solution is poured into a metal structure at a constant rate, hydrodynamic forces cause the metal structure to deform elastically into a certain shape. When the hydrodynamic force increases, the deformation of the metal structure increases and returns to normal when the hydrodynamic force is removed.

### 3.3. Fabrication of Three-Dimensionally Deformable Structures by Precision Electroforming

**Figure 7:**
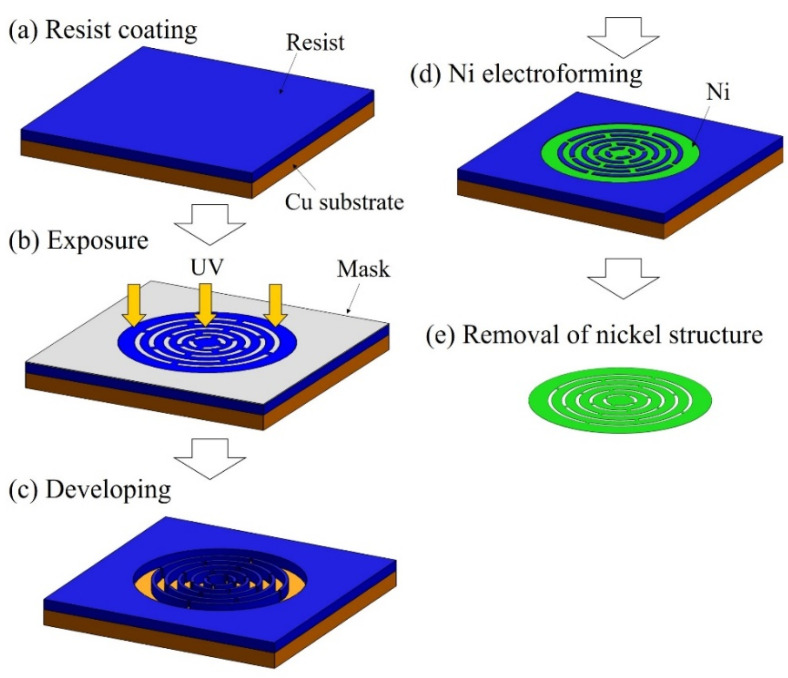
Schematic of the precision electroforming process. (**a**) A photoresist is applied on a Cu substrate. (**b**) Curing of photoresist by ultraviolet irradiation. (**c**) Mold creation by developing the photoresist. (**d**) Fabrication of Ni metal structures on Cu substrates by Ni electroforming. (**e**) Removal of the Ni structure to obtain a metal structure of the desired shape.

**Figure 8:**
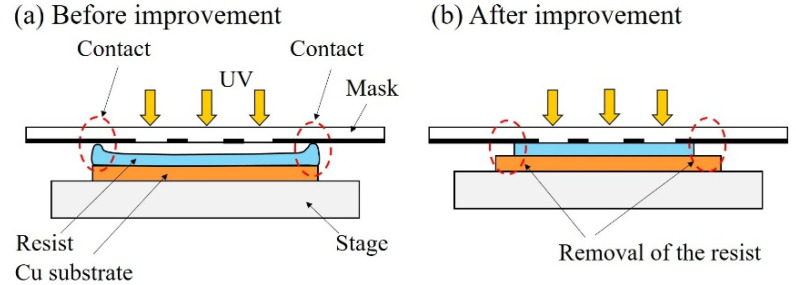
Improvement of the photolithography process. (**a**) After resist coating, the resist at the edge of the substrate is thick at approximately 60 μm. (**b**) Resist at the edges is physically removed after improvement. The thickness of the resist is uniform at 30 μm. There is no gap between the mask and substrate in the contact exposure.

**Figure 9:**
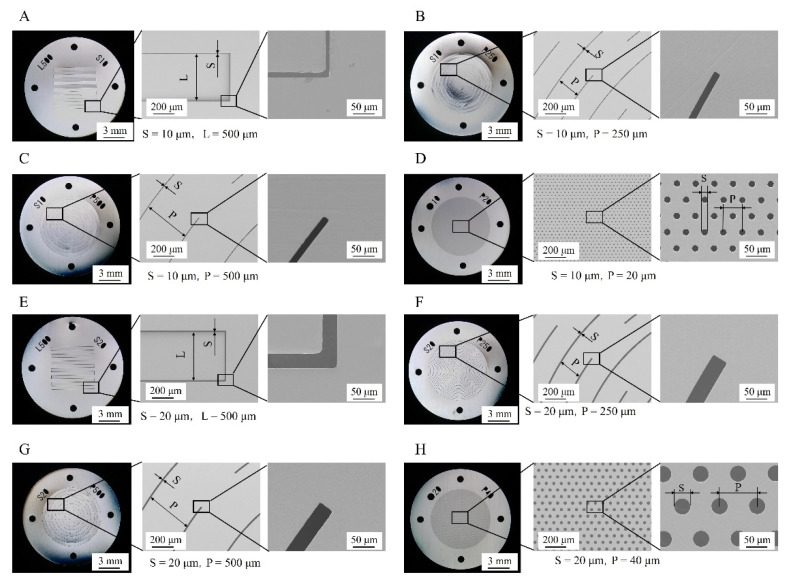
Photographs of the fabricated Ni structures. Eight types of Ni structures were fabricated precisely. Metal structures that deform three-dimensionally of Types (**A**–**C**) and (**E**–**G**) return to the original state even when deformed by hydrodynamic force. Metal structures of Types (**D**) and (**H**) are formed using the same processes and materials as metal structures that deform three-dimensionally and do not deform into three-dimensional shapes. All types appear warped under the light from the digital microscope, but the Ni structures are not warped after electroforming.

**Figure 10:**
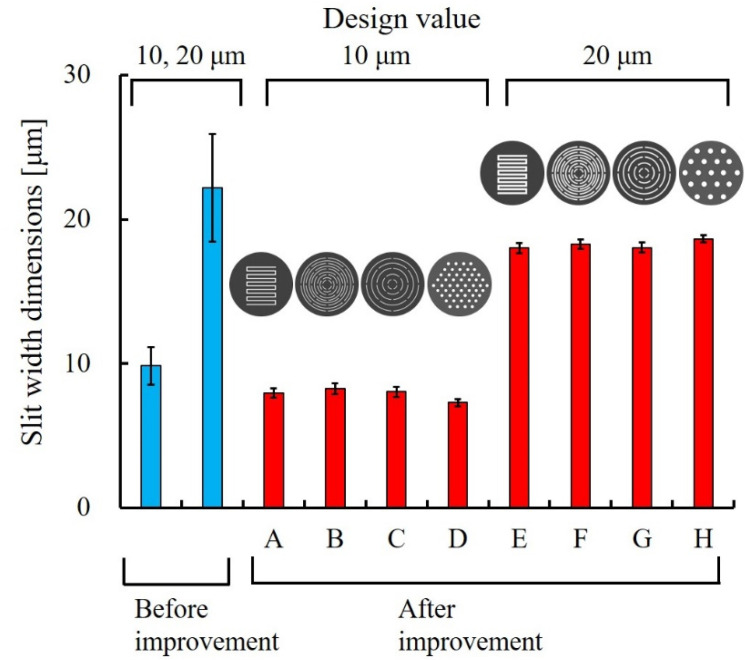
Metal-structure slit-width dimensions before and after improvement of the photolithography process. The dimensional variation decreases from σ = 3.74 to σ = 0.25 μm as a result of optimizing the photolithography conditions. The desired slit width is expected if the design value is adjusted.

**Figure 11:**
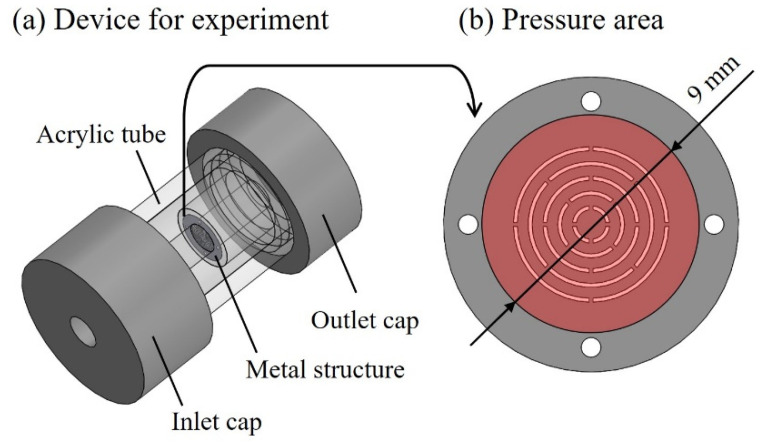
Overview of the device for the experiment. (**a**) The metal structure was fixed inside the device, and the hydrodynamic force was applied. The metal structure was fixed to an acrylic tube with an inner diameter of 9 mm. (**b**) Pressure was applied to the metal structure in a 9-mm-diameter area around the center.

**Figure 12:**
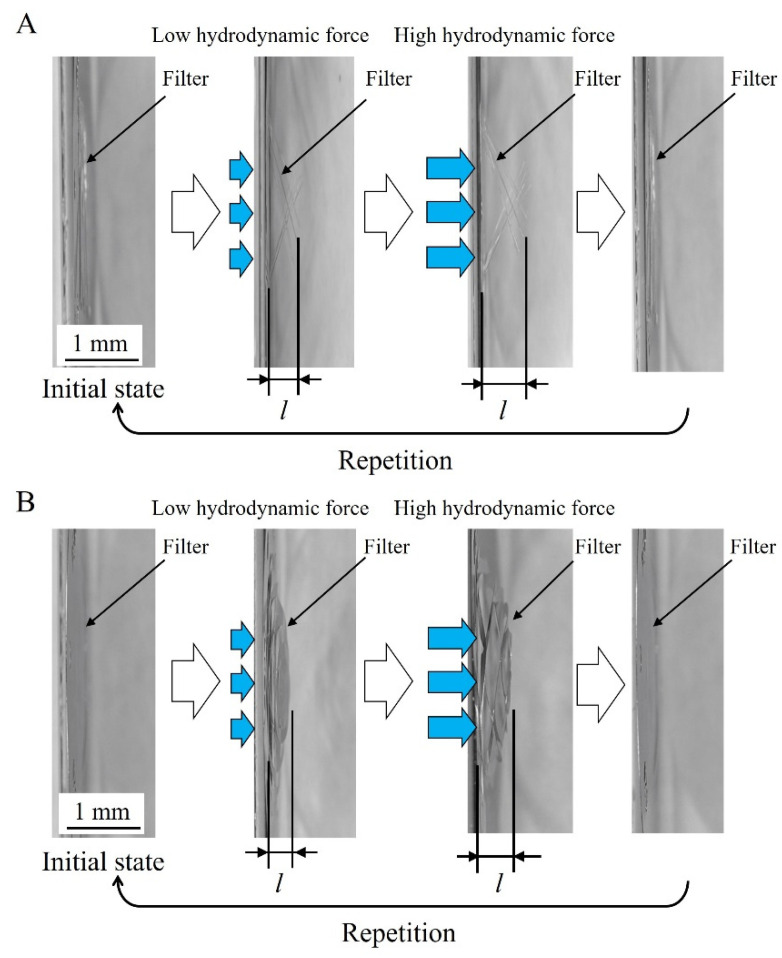
Photographs of three-dimensionally deformable metal structures. Type A is comb-shaped, and Type B is a metal structure with multiple arc-shaped slits. The deformation quantity l changes with the magnitude of the hydrodynamic force. When the hydrodynamic force is unloaded, the structure returns to its original state. The deformation of the Type A structure at a flow rate of 300 mL/h was approximately 110 μm, and that at a flow rate of 2400 mL/h was approximately 820 μm. The deformation of the Type B structure at a flow rate of 300 mL/h was approximately 40 μm, and that at a flow rate of 2400 mL/h was approximately 90 μm. The deformations of both structures increased linearly with increasing flow rate. In addition, a comparison of the simulated and measured deformation of the two metal structures under a flow rate of 300 mL/h revealed that Type A showed deformation of approximately 120 µm (simulation value), and the measurement result was 110 µm, while Type B showed deformation of approximately 40 µm (simulation value), and the measurement result was 40 µm. Under low flow-rate conditions, the simulated and measured results showed good agreement (Supplementary Materials Figure S2). There was no plastic deformation, even under conditions with a flow rate of 2400 mL/h. As there were no realistic conditions for exceeding 2400 mL/h, it was not possible to verify until plastic deformation occurred. In this study, as a limitation to elastic deformation, results were shown for conditions with a flow rate of 300–2400 mL/h.

Ni structures are intended to be applied to filters. It is possible to perform various chemical modifications on the surface of the Ni structure by adding Au plating, which can add functions to the structural surface. Typical examples include the modification of self-assembled monolayers (SAMs), frequently used in analytical chemistry and biochemistry, and antibody modifications for antigen-antibody interactions. Figure 7 shows the fabrication process of a metal structure with a fine pattern. In the photolithography process, a 30 µm positive liquid resist (AZ P4903, Merck Performance Materials Co., Ltd., Tokyo, Japan) was applied to a Cu substrate by a spin coater (MS-A150, Mikasa Co., Ltd.) (Figure 7a) and was exposed to light at 1.75 J/cm^2^ using a mask aligner (MA-20, Mikasa Co., Ltd.) (Figure 7b). A resist structure to serve as a mold was developed using an AZ 400 K Developer (Merck Performance Materials Co., Ltd.) (Figure 7c). To release the Ni structure from the Cu substrate, chromate treatment (2 g/dm^3^, 2 min, 30 °C) with potassium dichromate solution was applied on the Cu substrate before Ni electroforming. Next, using an electroforming bath, we fixed a Ni plate containing sulfur to the anode and a fabricated resist structure to the cathode to form a Ni structure by electroforming (Figure 7d). The resist structure was released using stripping solution (AZ Remover 700, Merck Performance Materials Co., Ltd.). By removing the resist structure, we obtained a metal structure of the desired shape by releasing the Ni structure from the Cu substrate with tweezers (Figure 7e). Figure 8 shows the improvements in contact exposure in photolithography. As the liquid resist used has a high viscosity of 1550 mm^2^/s, the resist was approximately 60 μm thick in the outer peripheral portion of the substrate post resist coating. The resist was physically removed using a 5 mm wide and 1 mm thick stainless-steel stick within 3 mm of the substrate edge, and the resist thickness was uniform at 30 μm. The removal of the excessively thick resist eliminated the gap between the mask and substrate, and the variation of the slit width decreased. Figure 9 shows photographs of the Ni structure fabricated by this process. We found eight types of structures, Types A–H, could be fabricated without defects, even in the slit portion or overall surface. Figure 10 shows the dimensional measurement results for the slit width of the Ni structure. By optimizing the photolithography conditions, the variation of the slit width improved from σ = 3.74 to σ = 0.25 µm. The slit width of Types A–D was 7.9 µm on average, with a design value of 10 μm, and the slit width of Types E–H was 18.3 µm on average, with a design value of 20 µm. In addition, the variation for all eight types was σ = 0.25–0.37 µm, and the fabrication was found to be precise. Although the slit width was approximately 2 µm smaller than the target value, it was expected that the desired slit width could be obtained by adjusting the design value. When fabricating a microstructure, the fact that the slit width becomes smaller than the target value is an advantage that may be adopted for various applications. The metal structure was fixed in the device shown in Figure 11, and hydrodynamic force was applied to demonstrate that the structure can deform from a two- to three-dimensional shape. Pressure was applied to the metal structure in a 9 mm diameter area around the center. Figure 12 presents a photograph of when a hydrodynamic force is applied to structures with a Type A comb shape and a Type B arc shape with multiple slits. The deformation amount of the Ni structures fabricated by this process changes according to the magnitude of the hydrodynamic force, and the structures return to their original shapes when the applied force is removed. Furthermore, if there is continuous flow at a constant rate, it is possible to maintain a constant deformation and the shape of the fluid. Incidentally, Types D and H, which have micropores formed in-plane, are self-supporting structures similar to the other structures. However, they do not deform into three-dimensional shapes under hydrodynamic forces.

**Figure 13:**
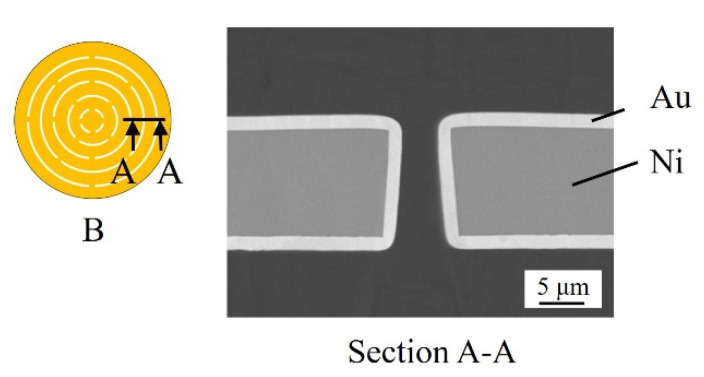
Cross-sectional photograph of a Type B three-dimensionally deformable metal structure (slit width: 10 μm) fabricated with a Ni electroforming thickness of 10 μm and Au-plating thickness of 2 μm. The tapered shape is due to the effect of diffraction of light during exposure.

Figure 13 shows a cross-sectional scanning-electron microscopy (SEM) image of a Type B metal structure fabricated using 10 µm and 2 µm as the target film thicknesses for Ni electroforming and Au plating, respectively. Type B is obtained with Au coating from the basic baths in Table 2. The digital microscopy and cross-sectional SEM images show that there are no pinholes or cracks on the surface of the metal structure, demonstrating the absence of structural defects. The Ni electroforming thickness is 10.1 μm, the Au-plating thickness is 2 µm, and the film thickness can be formed as designed. In addition, a structure with a high aspect ratio (film thickness/slit width = 3.9) could be fabricated with good precision using this process. Incidentally, the yield of the fabricated structure is 90%. The three-dimensionally deformable microstructure fabricated using the process developed in this study can be applied to bioMEMSs, analysis chips, and μTASs from the perspective of size. Therefore, these structures may be expected to be applied in the biomedical field.

## 4. Conclusions

The objectives of this study were to fabricate a metal structure that can be elastically deformed into a three-dimensional shape in response to hydrodynamic forces and to propose a fine pattern fabrication approach using precision electroforming. Physical properties, such as the film thickness, hardness, and slit width, were evaluated to fabricate the fine metal structures using this technique. Based on the results, we achieved σ = 0.49 µm with an average film thickness of 12.9 µm and σ = 0.25 µm with a hardness of 600 HV and slit width of 7.9 µm. The conditions for fabricating precision metal structures were also evaluated. When the intended metal structure was fabricated using the derived conditions, we successfully fabricated metal structures capable of dynamically deforming into three-dimensional shapes according to the hydrodynamic force. In this study, structures with hardness ranging 300–950 HV and flow velocities of 300–2400 mL/h were investigated, but they were within the elastic deformation range and did not deform plastically. As the type of metal and physical properties of metal structures fabricated using this technique can be varied, these structures can be applied to various fields and applications such as bioMEMSs and µTASs in the future.

## Figures and Tables

**Table 1:** Bath composition for Ni electroforming.

Ni(NH_2_SO_3_)_2_·4H_2_O	600 g/dm^3^
H_3_BO_3_	30 g/dm^3^
pH	4 ± 0.1
Temperature	40 ± 2 °C
Current density	5 A/dm^2^
Paddle agitation	Stroke 100 mm, 60 rpm

**Table 2:** Bath composition for Au plating.

Na_3_Au(SO_3_)_2_	21.6 g/dm^3^
Na_2_SO_3_	75.6 g/dm^3^
pH	8 ± 0.1
Temperature	45 ± 2 °C
Current density	0.2 A/dm^2^
Agitation	Stirring 300 rpm

## Data Availability

All the data generated or analyzed during this study are included in this published article.

## Supplementary Materials

The following supporting information can be downloaded at: https://www.mdpi.com/article/10.3390/mi13071046/s1. Figure S1: Simplified flexural evaluation of four types of Ni film samples (a)–(d). The Ni films in (a) and (b) were found to deform elastically and return to their original shapes without damage. The samples with Ni-P alloys in (c) and (d) cracked and broke while bending. In addition, they did not return to their original shapes. Figure S2: Simulation analysis results for the deformation of the Type A and B metal structures. The conditions used in this analysis are summarized in Table S1. The measured deformation and simulation results were compared. Under a flow rate of 300 mL/h, Type A showed a deformation (simulation value) of approximately 120 μm, and the measurement result was 110 μm, whereas Type B showed a deformation (simulation value) of approximately 40 μm, and the measurement result was 40 μm. Under a flow rate of 2400 mL/h, Type A showed a deformation (simulation value) of approximately 1080 μm, and the measurement result was 820 μm, whereas Type B showed a deformation (simulation value) of approximately 280 μm, and the measurement result was 90 μm. Thus, the simulation and measurement results agree well under the condition of a low flow rate. Table S1: Deformation analysis conditions.
