# Supplementary material for: Fabrication of Three-Dimensionally Deformable Metal Structures Using Precision Electroforming
Source: Micromachines (Basel). 2022 Jun 30;13(7):1046. doi: 10.3390/mi13071046 (PMC9315991; doi:10.3390/mi13071046)
Supplement: Supplementary file 1 [file micromachines-13-01046-s001.zip › micromachines-1765244-supplementary.pdf]

Supplementary Materials: Fabrication of Three-Dimensionally Deformable Metal Structures Using Precision Electroforming

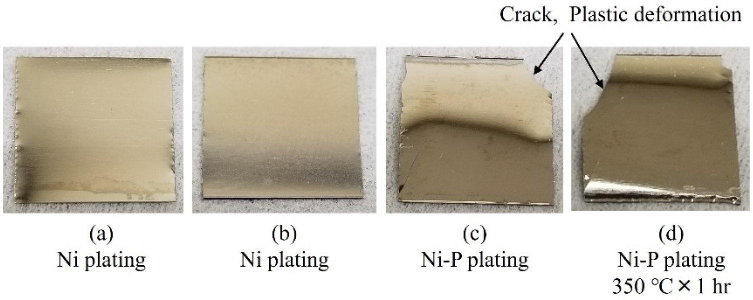

Figure S1. Simplified flexural evaluation of four types of Ni film samples (**a**–**d**). The Ni films in (**a**) and (**b**) were found to deform elastically and return to their original shapes without damage. The samples with Ni-P alloys in (**c**) and (**d**) cracked and broke while bending. In addition, they did not return to their original shapes.

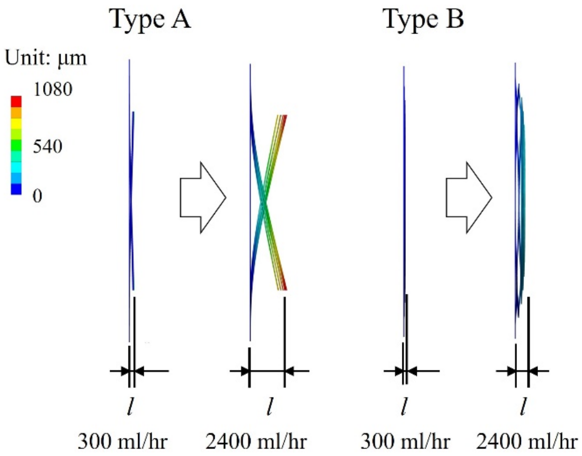

Figure S2. Simulation analysis results for the deformation of the Type A and B metal structures. The conditions used in this analysis are summarized in Table S1. The measured deformation and simulation results were compared. Under a flow rate of 300 ml/h, Type A showed a deformation (simulation value) of approximately 120 µm, and the measurement result was 110 µm, whereas Type B showed a deformation (simulation value) of approximately 40 µm, and the measurement result was 40 µm. Under a flow rate of 2400 ml/h, Type A showed a deformation (simulation value) of approximately 1080 µm, and the measurement result was 820 µm, whereas Type B showed a deformation (simulation value) of approximately 280 µm, and the measurement result was 90 µm. Thus, the simulation and measurement results agree well under the condition of a low flow rate.

Table S1. Deformation analysis conditions.

Item	Boundary Condition
Analysis model	Axis-symmetric
Young's modulus	200 GPa
Number of nodes	1,049,655
Number of elements	414,361
Element type	PLANE182 (2-D four-node structural solid)
